# A review of cytokine-based pathophysiology of Long COVID symptoms

**DOI:** 10.3389/fmed.2023.1011936

**Published:** 2023-03-31

**Authors:** Russell N. Low, Ryan J. Low, Athena Akrami

**Affiliations:** ^1^Retired, La Jolla, CA, United States; ^2^Gatsby Computational Neuroscience Unit, University College London, London, United Kingdom; ^3^Sainsbury Wellcome Centre, University College London, London, United Kingdom

**Keywords:** COVID-19, cytokines, Long COVID, neuroinflammation, post-acute sequelae of COVID-19, Myalgic Encephalomyelitis (ME), Chronic Fatigue Syndrome, Dysautonomia

## Abstract

The Long COVID/Post Acute Sequelae of COVID-19 (PASC) group includes patients with initial mild-to-moderate symptoms during the acute phase of the illness, in whom recovery is prolonged, or new symptoms are developed over months. Here, we propose a description of the pathophysiology of the Long COVID presentation based on inflammatory cytokine cascades and the p38 MAP kinase signaling pathways that regulate cytokine production. In this model, the SARS-CoV-2 viral infection is hypothesized to trigger a dysregulated peripheral immune system activation with subsequent cytokine release. Chronic low-grade inflammation leads to dysregulated brain microglia with an exaggerated release of central cytokines, producing neuroinflammation. Immunothrombosis linked to chronic inflammation with microclot formation leads to decreased tissue perfusion and ischemia. Intermittent fatigue, Post Exertional Malaise (PEM), CNS symptoms with “brain fog,” arthralgias, paresthesias, dysautonomia, and GI and ophthalmic problems can consequently arise as result of the elevated peripheral and central cytokines. There are abundant similarities between symptoms in Long COVID and myalgic encephalomyelitis/chronic fatigue syndrome (ME/CFS). DNA polymorphisms and viral-induced epigenetic changes to cytokine gene expression may lead to chronic inflammation in Long COVID patients, predisposing some to develop autoimmunity, which may be the gateway to ME/CFS.

## Highlights

- Long COVID patients include millions of people worldwide, and persistent symptoms following COVID-19 can continue for months.
- Varied and relapsing symptoms of Long COVID can be attributed to elevated peripheral and central cytokines, generated by an abnormal immune response.
- Chronic inflammation linked to immunothrombosis with microclot formation leads to decreased tissue perfusion and ischemia.
- CNS effects may be due to direct viral invasion, indirect immune response, or immunothrombosis.
- Dysregulated activation of brain microglia, due to neuroinflammation, can cause centrally mediated symptoms.
- Upregulation of the p38 MAPK pathway by SARS-Cov-2 can be a possible mechanism by which the virus increases cytokine production.
- Progression to myalgic encephalomyelitis/chronic fatigue syndrome (ME/CFS) and dysautonomia occurs in some patients and could involve development of autoimmunity.

## 1. Introduction

Long COVID, or Post Acute Sequelae of COVID-19 (PASC), is a debilitating illness with symptoms lasting more than 12 weeks following COVID-19 infection (1). Long COVID was first characterized in the COVID-19 Prolonged Symptoms Survey – Analysis Report of May 11, 2020, which described 640 patients with a protracted clinical course greater than 2 weeks (2). The report showed that, among the participants, the chance of full recovery by day 50 was less than 20%. In another study, 85% of patients who had symptoms 2 months after the initial infection continued to experience symptoms at 1 year follow up (3). The actual number of people worldwide struggling with Long COVID has not been measured systematically. However, current estimates suggest between 10% and 40% of people infected with COVID-19 have prolonged symptoms (4–7). As of January 2023, the over 670 million cases of COVID worldwide translate to at least 67 million persons with Long COVID. Among patients hospitalized for COVID-19 infection, an even larger number (up to 90%) can suffer from symptoms 8–12 weeks after admission (8–10). The incidence of Long COVID is reduced in individuals who have been vaccinated (11, 12).

Long COVID patients suffer from a plethora of symptoms, impacting their quality of life and ability to work (13). Early reports described the top 10 reported symptoms as shortness of breath, chest tightness, mild-to-moderate fatigue, chills or sweats, body aches, dry cough, fever (98.8–100°F, 37.2–37.8°C), headache, and cognitive/concentration challenges termed “brain fog” (2). Over 200 symptoms have since been described, affecting multiple organ systems (13). For example, cardiopulmonary involvement can produce chest pain, palpitations, cough and dyspnea. Gastrointestinal tract involvement (13, 14) can produce abdominal pain, and nausea while solid abdominal organ involvement of the pancreas, liver, spleen and kidney may result in end organ injury. Recognized vascular endothelial injury (15, 16) can lead to coagulopathy, pulmonary emboli and strokes. A host of neurological symptoms can result in cognitive impairment, brain fog, sleep and memory disturbances, tinnitus and vertigo (13). Involvement of the reproductive system may lead to erectile dysfunction and irregular menstruation (13). Finally, the immune system as a cause or casualty of Long COVID can result in autoimmune diseases (17). At 6 months post-infection, the most commonly reported Long COVID symptoms primarily include a combination of systemic and neurological issues such as fatigue, cognitive dysfunction, and post-exertional malaise (PEM)—wherein symptoms are markedly exacerbated by physical or mental stress/exertion, with the onset measured in hours to days following the activity (13).

Analyzing the pattern of symptoms, in terms of the underlying pathophysiology on cellular and macromolecular levels, will shed light on the Long COVID patients’ clinical plight. Questions persist about the pathophysiology of their symptoms and their eventual outcome (17). The multifactorial processes underlying Long COVID may include end organ disease, alterations in immune-inflammatory response, mitochondrial energy production, oxidative stress, and aberrant coagulation with microthrombosis. The relative contributions of each process may be different in individual patients. However, these are interrelated processes which all involve inflammatory cytokines (18). In this article, we propose a description of the pathophysiology of Long COVID symptoms based on inflammatory cytokines and a dysregulated immune-inflammatory response, and we review the possible pathways involved.

Viral infections incite a pro-inflammatory response with increased production of cytokines and chemokines (19, 20). Cytokines are small proteins involved in cell signaling. They are secreted by numerous cell types, including endothelial cells, macrophages, monocytes, and lymphocytes, and can act in an autocrine, paracrine or endocrine manner. Cytokines can be broadly categorized into different families, including interferons, interleukins, colony-stimulating factors, and tumor necrosis factors (21). Chemokines recruit inflammatory cells as part of the normal immune response (21).

Several studies have shown that both mild and severe cases of COVID-19 can result in a hyper-inflammatory response characterized by elevated levels of numerous cytokines, including IL-6, IL-8 and TNF-α (22–24). While these proinflammatory cytokines are expected to be induced upon viral infection, SARS-CoV-2 encodes several proteins that specifically evade initial type I interferon response (25, 26), resulting in a prolonged and, in certain cases, dysregulated cytokine response. A recent study shows an abnormal diffuse inflammatory cytokine profile that persists in Long COVID patients for at least 8 months, not found in asymptomatic COVID-19 survivors (27).

Studies of Long COVID patients have shown persistent deregulation of a broad range of cytokines long after infection. Specifically, it is shown that IL-1β, IL-6 and TNF-α remain elevated in Long COVID - PASC (28). This is despite the fact that peripheral blood cytokines do not need to be elevated, since they function by acting locally rather than by flowing through the blood (29). Nevertheless, persistent immune activation has been suggested to be associated with ongoing symptoms following COVID-19 (30, 31).

An abnormal immunologic response to the virus can result in an uncontrolled systemic inflammatory response with more severe infections (32). This “Cytokine Storm,” has gained widespread attention as it is associated with severely ill patients with COVID-19 pneumonia who show a progression of symptoms (23). Acute Respiratory Distress Syndrome (ARDS), requiring mechanical ventilation and ICU admission, results in a mortality rate approaching 40%–50% in COVID-19 patients (33, 34). Uncontrolled overproduction of pro-inflammatory cytokines and chemokines produces the cytokine storm, resulting in systemic inflammation, ARDS, and multiple organ failure (21, 35–37). The relationship between cytokine production and tissue damage is not necessarily causal and is clearly bidirectional. While also involving cytokines, the situation and clinical course for the Long COVID patient is quite different.

## 2. The cytokine release syndrome

Cytokines are small endogenous proteins that mediate communication between cells, particularly cells of the immune system. Cytokines control the growth and activity of immune cells and blood cells and are essential for directing the body’s immune-inflammatory responses and maintaining homeostasis.

While the focus on the “cytokine storm” is clinically relevant in the severely ill COVID-19 patients, the term ignores the role cytokines can play in the symptoms of less severe disease, including those of Long COVID patients.

**Figure 1:**
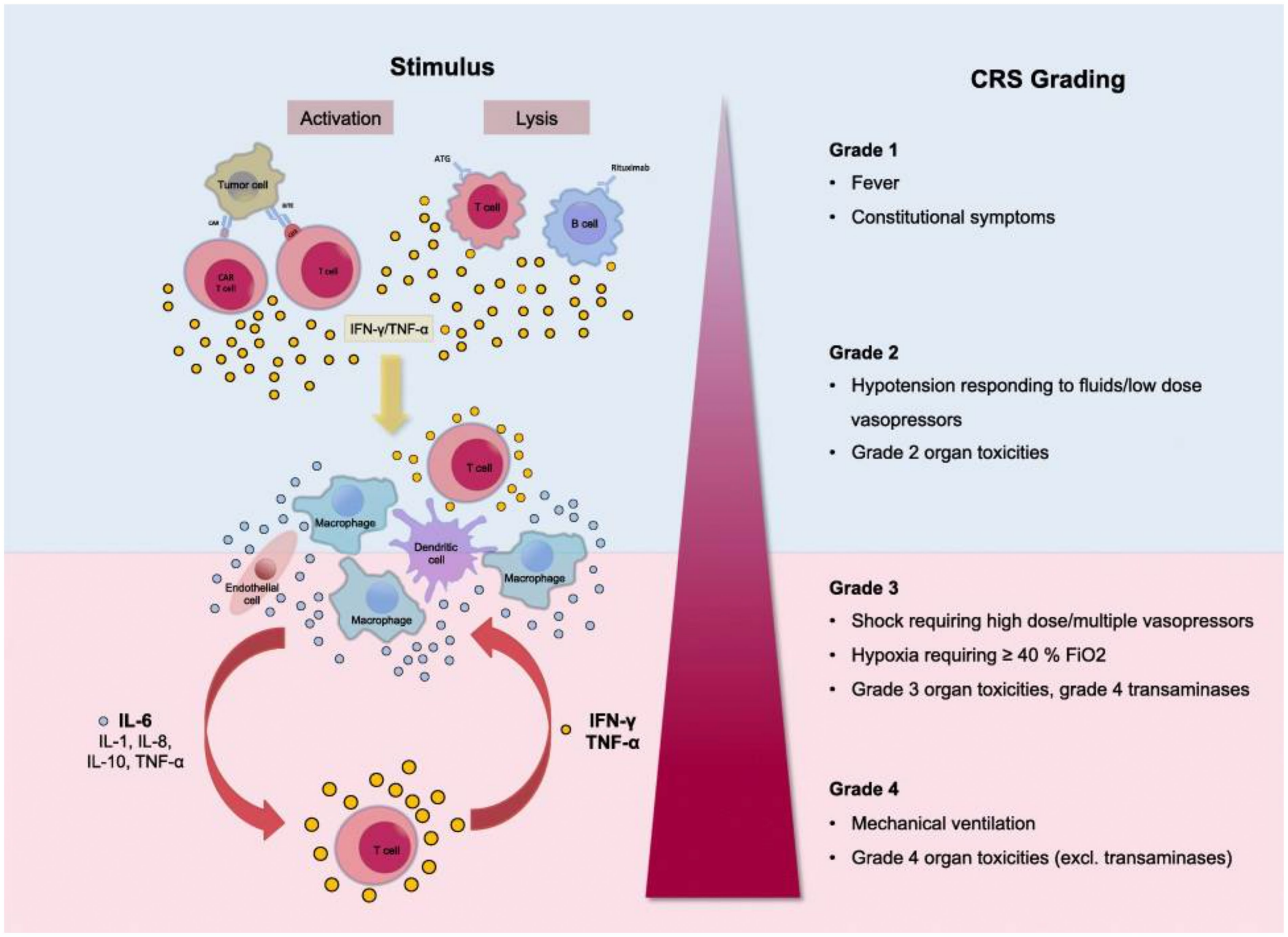
Grading of the Cytokine Release Syndrome. Activation of T cells induces a release of IFN-γ or TNF-α. This process activates macrophages and other immune cells, releasing proinflammatory cytokines. Large amounts of IL-6 are released, which in a positive feedback loop activates T cells and other immune cells. Reproduced from “Cytokine Release Syndrome” by AJ Shimabukuro-Vornhagen, P Gödel, M. Subklewe, HJ Stemmler, HA Schlößer, M Schlaak, et al. 2018 Immunother. Cancer 56 (http://creativecommons.org/licenses/by/4.0/).

Instead, we believe that the Cytokine Release Syndrome (CRS) description provides a more balanced presentation of the spectrum of symptoms in patients with clinical manifestations of elevated cytokine production (38, 39). CRS is a systemic inflammatory response triggered by infections, drugs, antibody-based immunotherapies, chemotherapeutic agents, and graft-*vs*-host disease (both acute and chronic) (38) (Figure 1).

Clinical presentation ranges from mild flu-like symptoms to life-threatening “cytokine storm.” The CRS grading system includes Grade 1 with fever and mild constitutional symptoms, Grade 2 with hypotension responding to fluids and vasopressors, Grade 3 with shock, hypoxia, and Grade 4 with respiratory failure and ARDS requiring mechanical ventilation, disseminated intravascular coagulation (38, 40). CRS is a multiorgan process affecting the lungs, CNS, spleen, gastrointestinal tract, kidneys, hepatobiliary tree, heart, musculoskeletal system, white cells, and platelets.

In general, Grade 1 CRS with fever, fatigue, headache, rash arthralgia, and myalgia correlates with the symptoms of Long COVID patients (see the section Long COVID Symptoms and Cytokine Release Syndrome Model). As with the more severe CRS grades, Grade 1 symptoms result from proinflammatory cytokine release from activated macrophages and white cells, initiating the inflammatory cascade.

These cytokines and chemokines recruit immune cells (41), which then secrete further cytokines producing a positive feedback loop. While this process occurs in the body’s normal inflammatory response, in the CRS, loss of regulation of the inflammatory cascade can result in an uncontrolled destructive process (21, 42). The cytokine cascade’s autoimmune inflammatory effect can be responsible for more damage than is produced directly by the SARS-CoV-2 virus (43).

There are critical inter-individual differences in the cytokine pathway. The COVID-19 comorbidities are characterized by low-grade inflammation and elevated cytokine levels (44). For example, the comorbidities of aging (45), obesity (46), diabetes mellitus (47, 48), and hypertension (49) are associated with increased proinflammatory cytokines and a worse COVID-19 prognosis (50).

## 3. Long-COVID symptoms and cytokine release syndrome model

With this knowledge of the effects of cytokines in COVID-19 patients, how can one explain the varied and relapsing symptoms of Long COVID patients? This section will review the evidence linking elevated cytokines to various symptoms commonly experienced in Long COVID patients.

### 3.1. Dysregulated peripheral immune response

Macrophages are the first line of innate immunity, producing various inflammatory cytokines and chemokines. Polarization between a reparative M2 and proinflammatory M1 state occurs, which can become dysregulated to favor proinflammatory cytokine release (Figure 3). This macrophage activation syndrome (MAS), present in systemic inflammatory diseases like systemic lupus erythematosus, juvenile rheumatoid arthritis and Still Disease, can result in life-threatening inflammation. Diminished function of NK cells and cytolytic CD8 T cells prolongs and amplifies the inflammatory response (51). In COVID-19, pathologic hyperinflammation due to dysregulated macrophages leads to prolonged inflammation and host damage (42, 51, 52).

### 3.2. A mechanism for CNS mediated symptoms

**Figure 2:**
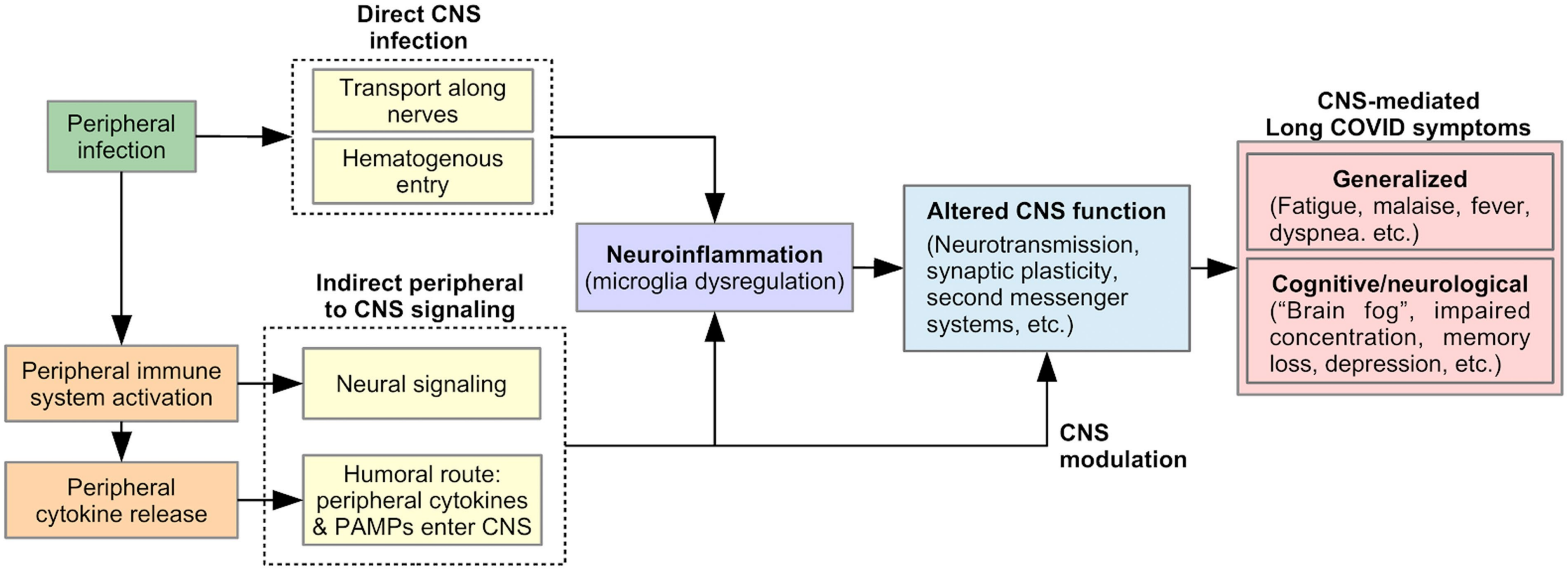
Proposed model of pathophysiology underlying CNS-mediated Long COVID symptoms.

Many Long Covid symptoms may be centrally mediated by the brain, including generalized symptoms such as fatigue, malaise, fever, and dyspnea (53), as well as cognitive and neurological symptoms such as “brain fog,” concentration difficulty, memory loss, dizziness, and disequilibrium (2, 6, 54). Viral-induced CNS changes can explain the persistent and relapsing course of these symptoms in Long COVID. We describe two pathways that could produce such changes (Figure 2): (1) neuroinflammation, triggered by direct CNS infection or signals from the peripheral immune system, and (2) modulation of CNS function by a persistently activated peripheral immune system.

#### 3.2.1. Direct viral invasion of the CNS

SARS-CoV-2 is neuroinvasive, neurotropic, and neurovirulent (55). Viral proteins have been found in the brainstem and cranial nerves in autopsies (56). The route of SARS-CoV-2 entry into the CNS is currently unknown, but could include: (1) Transport along cranial and peripheral motor, sensory, or autonomic nerves (e.g., olfactory to CNS invasion) (55, 57, 58). Here, the virus invades nerve endings and is actively transported within neurons to the brain. (2) Alternatively, hematogenous spread could occur, in which the virus passes from alveolar epithelial cells or endothelial cells of other organs into the blood circulation (55). The virus penetrates the Blood Brain Barrier (BBB) through points of weakness (e.g., circumventricular organs and the choroid plexus), or by damaging the vascular endothelium of the BBB, resulting in neuroinvasion (55, 57). Neuroinvasion can trigger neuroinflammation, thereby altering CNS function and producing Long COVID symptoms, as discussed below (Figure 2).

#### 3.2.2. Indirect immune-mediated viral effects on the CNS

While direct CNS viral infection is possible, indirect CNS involvement through a viral-mediated immune response is equally likely (56, 59). As part of the innate immune response, proinflammatory cytokines are released by activated peripheral immune cells in response to pathogen-associated molecular patterns (PAMPS) detected by pattern-recognition receptors (PRRs). Peripherally released cytokines act on the brain *via* two pathways of communication: (1) a neural route *via* primary afferent neurons, including the vagus nerve, innervating the site of the infection; (2) a humoral route, in which peripheral cytokines or PAMPS cross the BBB at the circumventricular organs and choroid plexus and stimulate the production of proinflammatory cytokines by brain immune cells (60, 61). As discussed below, these signals from the peripheral immune system to the CNS could lead to Long COVID symptoms by triggering neuroinflammation and/or by directly and persistently modulating CNS function (Figure 2).

### 3.3. Neuroinflammation and microglial cell dysregulation

Direct or indirect viral effects on the CNS can trigger neuroinflammation—a chronic immune response within the brain involving long-term activation of microglia, the local release of inflammatory cytokines, and resulting oxidative stress (62, 63). Neuroinflammation has been implicated in the pathogenesis of many neurodegenerative (64) and psychiatric diseases (65), and can also be triggered by viral infection (62). In a recent study, pronounced neuroinflammatory changes were found in the brainstem of post-mortem brain autopsies in COVID-19 patients (56). In these patients, evidence of SARS-CoV-2 viral proteins was found in over half of patients in the brainstem and cranial nerves. However, neuropathological changes were not associated with the presence of the virus. Instead, the CNS damage and neurological manifestations (66) were attributed to cytokine-mediated neuroimmune stimulation, also indicated by the presence of neuroinflammation in the ocular vitreous cavity of persons who recovered from COVID-19 (67).

**Figure 3:**
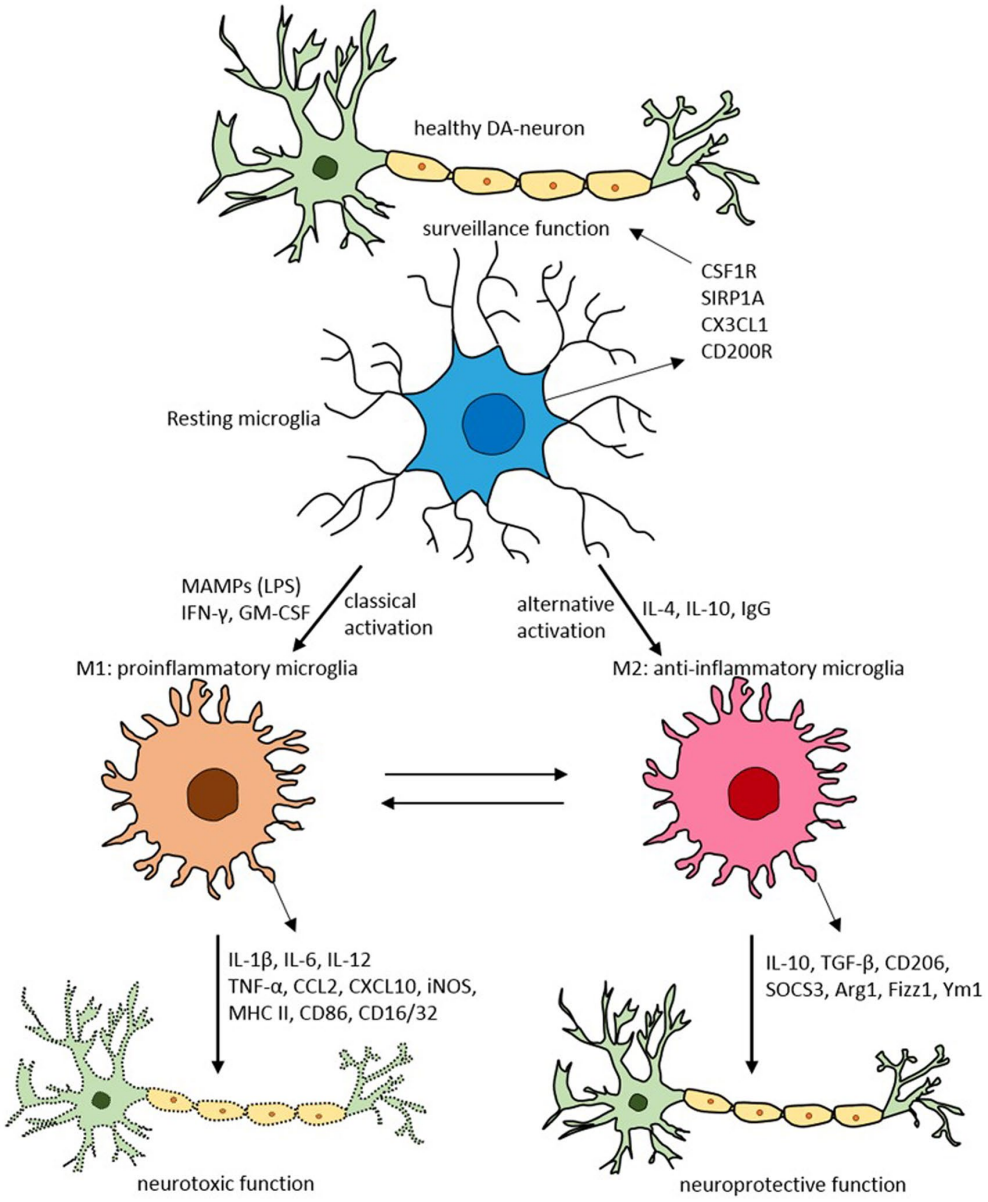
Schematic of microglial polarization states and functions. Reproduced from “Targeting microglial activation states as a therapeutic avenue in Parkinson’s disease” by SR Subramaniam and HJ Federoff. (http://creativecommons.org/licenses/by/4.0/).

Glial cells, such as astrocytes and microglia, play an essential role in brain neuroinflammatory insults (68). Microglia, the resident brain macrophages, acquire different functional phenotypes in response to their environment, alternating between a quiescent reparative M2 state (anti-inflammatory) and an activated proinflammatory M1 state (69, 70) (Figure 3).

Usually, the system is well regulated. However, with aging, chronic infection, or stress, microglia can become dysfunctional (72, 73). These dysregulated microglia are hyperreactive to signals from the peripheral immune system, producing an exaggerated and prolonged central cytokine response to an otherwise mild immune challenge (74, 75). The primed microglia then become resistant to normal regulation, failing to revert to the quiescent state after inflammation resolution (76). The dysregulation of microglia has been implicated in the pathophysiology of numerous neurodegenerative disorders (77).

### 3.4. Cytokine modulation of the CNS

In addition to neuroinflammation, circulating cytokines can modulate systemic functions by direct action on the brain (78, 79). This modulation is known to occur in response to viral infection. For example, even non-neurotropic viruses that do not invade the brain can cause CNS effects, triggered by a peripheral immune response (80). Human and animal studies have shown that increases in circulating cytokines can induce typical “sickness behaviors” (79, 81–83), including decreases in general activity, exploratory behavior, social and sexual interaction, food and water intake, and preference for sweets (anhedonic behavior), as well as altered sleep and impaired learning. Injection of IL-1ra (an inhibitor of pro-inflammatory IL-1) can suppress hypothalamic–pituitary–adrenal (HPA) axis activation and the associated sickness behaviors (sleep and behavioral changes) that were induced by systemic administration of an endotoxin (84).

Evidence shows that cytokines (e.g., IL-1β, IL-6, and TNF-α) play a role in complex cognitive processes *via* neuromodulation (85, 86), second messenger systems in neurons (87, 88), neurogenesis (86), and modification of synaptic plasticity (85). Cytokines can also induce pyrogenic responses in the brain by the synthesis of prostaglandins (89), and can additionally influence many neuroendocrine systems, the most prominent of which is the HPA-axis (89, 90). Cytokines can also influence the synthesis, secretion, and reuptake of many central neurotransmitters, including noradrenaline, dopamine, glutamate, serotonin, GABA, and acetylcholine, as well as the expression of several neuropeptides in different brain areas (89–92). The alterations in neurotransmission may lead to depression, anxiety, and mental sluggishness or “brain fog” (93, 94). Overexpression of cytokines has been associated with neuropsychiatric disorders such as depression (95–97). IL-6 has recently been implicated as a mediator in COVID-19 neuropsychiatric symptoms (98).

In summary, a peripheral inflammatory response may lead to cytokine release and mobilization of immune cells, both of which have CNS effects (94, 99). SARS-CoV-2 can affect the CNS *via* proinflammatory cytokines, producing neurological symptoms (99). Such “sickness behavior” usually resolves once the acute illness terminates. However, subsequent neuroinflammation and oxidative stress can produce prolonged sickness behavior, cognitive deficits, and increased sensitization to intrinsic and extrinsic stress (75). In Long COVID, dysregulation and chronic activation of cytokine signaling, as discussed later, can cause prolonged CNS effects. This model of dysregulated, activated, and primed brain cells and microglia, responding to peripheral or central cytokines, can explain the chronic and relapsing clinical course of many Long COVID symptoms.

### 3.5. Mild constitutional symptoms

Almost all Long COVID patients experience systemic symptoms (13). The constitutional symptoms of fever, chills, headache, arthralgia, and mild dyspnea can be attributed to cytokine release (38, 100). These symptoms of “sickness behavior” are mediated by the brain which recognizes proinflammatory cytokines as signals of sickness (see CNS Mediated Symptoms). The peripheral innate immune response to pathogen-associated molecular patterns (PAMPS) produces cytokines (IL-1, IL-6, and TNF-α) (101). Most of these proinflammatory cytokines are pyrogenic (IL-6, IL-1, IFN-γ, and TNF-α. TNF-α can have both pyrogenic and antipyretic properties) and their systemic circulation can induce a febrile response (102–104).

### 3.6. Neuropathic pain

Paresthesia reported in Long COVID patients (105) can be related to cytokines. In patients with diabetic neuropathy, neuropathic pain correlated with increased levels of IL-6 and IL-10 for large nerve fiber damage (106). Patients with polyneuropathies show elevated serum cytokines IL-8, TNF-α and lower expression of IL-10 (107). Disease duration positively correlates with IL-6 gene expression (107).

Post-infectious neuropathies are precipitated by an autoimmune process in which antibodies to the infectious agent cross-react with peripheral nerve myelin (108). Like other neuropathies, the process is directly related to cytokines (109), which are also a therapeutic target (110).

### 3.7. Extreme fatigue

Fatigue is highly prevalent among Long COVID patients (13, 111). Post-viral fatigue is well described (112). Extreme physical fatigue can have two components: actual “muscle fatigue” and “central fatigue,” related to abnormalities in deep brain circuits involved in motor planning and execution (113). For myalgic encephalomyelitis/chronic fatigue syndrome (ME/CFS) patients and perhaps for Long COVID patients, the fatigue can originate centrally with decreased basal ganglia function, including psychomotor slowing (114, 115). With chronic inflammation, cytokines target the basal ganglia and dopamine function, producing persistent fatigue (116).

Cytokines cause neurotransmitter release in the brain, including serotonin, producing extreme central fatigue (117). In healthy patients, the administration of IL-6 and IL-1β produces fatigue, as well as sleep disturbances, and difficulty concentrating (118). In people with post-infection fatigue, recovering from West Nile virus, elevated levels of both proinflammatory and antiviral cytokines (IL-2, IL-6, IFN-γ, IL-10, among others) were detected (112). In patients with rheumatoid arthritis and systemic lupus erythematosus, fatigue improved with anti-IL-6 medications (119, 120). Viral-induced mitochondrial dysfunction leads to decreased ATP and increased cytokines IL-1β and IL-18 through activation of inflammasomes (121, 122). A similar mechanism can be at play in COVID19, where mitochondrial hijacking by SARS-COV-2 is suggested (123, 124).

### 3.8. Post-exertional malaise

Post-exertional malaise (PEM), or post-exertional symptom exacerbation (PESE), is characterized as worsening or relapse of symptoms after physical or mental activity. PEM is one of the most striking and demoralizing symptoms of Long COVID, reported by nearly 90% of patients (13). In the majority, one to two days after mild–moderate exercise, Long COVID patients experience marked worsening of symptoms with extreme fatigue, malaise, fever, shortness of breath, and neurological deficits.

Dysfunctional mitochondria can partially mediate PEM in muscles and brain microglia with less efficient glycolysis. Subsequent lactic acidosis with increasing reactive oxygen species (ROS), and elevated cytokines results in exercise-induced peripheral and central fatigue (125, 126).

For decades, exercise physiologists have known that exercise induces cytokine release (127). Suzuki (128) described a 2-15-fold increase in serum cytokines following maximal exercise. Following a marathon race, serum IL-6 levels increased 100-fold (128). Modest increases of IL-6 are expected after exercise of low-to-moderate intensity or intermittent physical activity of a shorter duration (129). Thirty minutes of acute exercise resulted in a 165% rise in IL-6 and a 32% rise in IL-8 in endurance-trained and sedentary young men (130).

Exercise-induced IL-6 responses vary with exercise intensity and duration, while exercise mode has little impact (131). Plasma IL-6 peak occurs as soon as the activity ends, returning to baseline after a few hours of recovery (127, 132). The exercise-induced cytokine release was more marked in the evening compared to the morning (133). In ME/CFS, exercise can induce significant changes in cytokine profile that are still observed 18 h following the exertion (134). Also, in ME/CFS patients experiencing PEM after moderate exercise, the severity of symptom flare is linked to cytokine activity, with increased circulating cytokines (135).

We argue that for COVID-19 patients, exercise-induced cytokines may trigger a recurrence of symptoms by reinitiating the same feedback loop pathways that caused the initial symptoms. Essentially, symptoms’ original occurrence and PEM-induced recurrence could both be due to peripheral and central cytokine cascades.

#### 3.8.1. Psychological stress precipitates cytokine release

Intense mental concentration precipitates a recurrence or worsening of symptoms in Long COVID patients identical to physical stress. In Davis et al., 60% of participants reported relapses, triggered by stress (13). It is known that social, psychological, or mental stress is also associated with cytokine release (136). A meta-analysis of 34 studies found increases in circulating IL-6, IL-1β, IL-10, and TNF-α and stimulated IL-1β, IL-4, and interferon-γ in response to acute psychological stress (137). The peak IL-6 level was at 90 min post-stress with a > 6-fold increase over baseline.

#### 3.8.2. The onset of symptoms post exertion

The delay in onset of symptoms following physical or mental stress is also predictable because the cytokines do not cause the symptoms; they just start the process of immune system recruitment and activation that leads to the symptoms. Cancer immunotherapy drugs produce the CRS with an onset of symptoms within a few days of the drug administration (38). This timing correlates with the delayed onset of Long COVID symptoms after exercise.

### 3.9. Gastrointestinal tract symptoms

More than 85% of Long COVID patients experience gastrointestinal symptoms (13). In COVID-19, recent research has shown that the infection disrupts the gut microbiome, allowing secondary pathologic bacteria to colonize the gut (138). Gut microbiome alterations have been documented in COVID-19 (139). Cytokines predispose to dysregulation of the microbiome (dysbiosis), consequently altering intestinal permeability, referred to as the “leaky gut,” allowing the bacteria to spread from the gut to the bloodstream. Entry of SARS-COV-2 into intestinal cells leads to down regulation of ACE2 receptors and subsequent decreased barrier function (140).

Ten percent (2%–50%) of COVID-19 patients report diarrhea, sometimes alternating with constipation (141). SARS-Cov-2 is present in feces in 55% of patients (142, 143). Wang et al. reported a 25-day median duration of fecal viral shedding with only 25% reporting diarrhea and 1% with nausea and vomiting. No significant differences were observed in GI symptoms between patients with positive and negative fecal viral tests (143). In patients with Long COVID, alterations in the gut microbiome have been observed (144).

The GI symptoms in Long COVID patients can be due to cytokines (145, 146) and associated cytokine-linked changes in the gut microbiome (145, 146). Diarrhea in systemic infections without intrinsic GI tract disease is well known. Pathogenesis may be related to cytokines, including IFN-γ, IL-6, and IL-10 (147). In patients with irritable bowel syndrome, pro-inflammatory cytokines, IL-6, and TNF-α, are elevated (148).

The microbiota-gut-brain axis can impact cognition and psychiatric symptoms, and lead to neurodegenerative diseases (140, 149). The interaction of gut microbiota with the brain may be through chronic inflammation, with cytokines altering maintenance of the blood brain barrier (150).

### 3.10. Arthralgia

Arthralgia has been described in 15% of coronavirus patients (151). More than 90% of Long COVID patients report musculoskeletal issues, including joint pain (~30% of LC patients) (13). Rheumatologists have noted that the pattern of elevated pro-inflammatory cytokines in COVID-19 are similar to those in patients with rheumatoid arthritis (151).

Possible mechanisms for the pathophysiology of joint pain/stiffness include the SARS-V2 activation of Toll-like receptors and the complement system leading to inflammation and autoantibody formation (152). Rheumatologic and autoimmune manifestations of Long COVD may be due to molecular mimicry, bystander killing, and viral persistence with polyclonal activation during longstanding presence of the virus resulting in immune mediated injury (153).

### 3.11. Ophthalmic problems

Ophthalmic manifestations of COVID-19 reported in hospitalized patients, include conjunctival hyperemia, and chemosis, epiphora, and increased secretions (154, 155) (156). A third of Long COVID patients report vision or other eye related issues (13). Ocular mast cells produce the inflammatory cytokines resulting in conjunctival hyperemia and chemosis (157). Visual and ocular disturbances in Long COVID patients may be similar to those reported in ME/CFS, including blurred vision, difficulty focusing, difficulty tracking lines of print, diplopia, light intolerance, and problems with peripheral vision (158). Evidence of inflammatory cells in the vitreous cavity have been found in patients recovered from COVID-19 (67).

The neuropathological mechanisms of SARS-CoV-2 infection involve multiple peripheral and cranial nerves, serving as conduits for the virus to gain access to the brainstem and olfactory cortex and to subsequently spread within the brain and to peripheral organs *via* cranial nerves (159). An inflammatory process in the central nervous system often manifests as optic neuritis (160). Associated neurological symptoms are seen in 25% of patients (161).

Cytokine-induced brainstem neuroinflammation could produce some of these visual problems without direct optic infection. Ocular tropism of SARS-CoV-2 was confirmed in a mouse model manifesting retinal inflammation with production of pro-inflammatory cytokines in the eyes of intranasally (IN)-infected mice (162). Finally, an autoimmune mechanism may also be at play in patients with COVID-19 optic neuritis due to anti-MOG antibodies (myelin oligodendrocyte glycoprotein) (163, 164).

### 3.12. Thrombotic complications of COVID-19: Relationship to cytokines

The processes of inflammation and thrombosis are intimately connected. Dysregulation can produce immunothrombosis and thrombinflammation, resulting in tissue injury from ischemia due to micro and macrovascular clotting (165).

The prothrombotic complications of SARS-CoV-2 became evident with reports of small vessel cerebral infarctions in young patients (166). Autopsies described the lungs and other organs full of micro clots (167). Myocardial infarctions, deep vein thrombosis, and systemic arterial emboli describe a systemic thrombotic process initiated by the SARS-CoV-2 virus. In a recent study, 31% of 184 COVID-19 ICU patients had thrombotic complications (166).

Inflammation-induced thrombosis is well known and, in addition, coagulation also intensifies inflammation through release of proinflammatory cytokines and growth factors, creating a vicious circle (168). The pathophysiology of thrombosis in COVID-19 may involve direct vessel endothelial injury by the SARS-CoV-2 binding to the ACE2 receptor (169). Also, pro-inflammatory cytokines increase blood viscosity (170–172), and specific cytokines, including interferon-γ, IL-6, IL-8, TNF-α, chemokine-ligand-2, IL-17A, IL-9, IL-1β, and growth-factor, have a prothrombotic effect (173, 174).

SARS-Cov-2 binds to platelet ACE2 receptors, leading to hyperactivation and platelet aggregation (175). Furthermore, spike protein promotes the release of coagulation factors and inflammatory cytokines from platelets (175). In turn, cytokines and erythropoietin potentiate platelet maturation. Platelet-producing megakaryocytes are found in unusual anatomic locations at autopsy of COVID-19 patients (176). SARS-CoV-2 induces gene expression leading to platelet hyperreactivity, contributing to COVID-19 pathophysiology (177). Critical interactions between inflammatory and thrombosis pathways, leading to SARS-CoV-2 induced vascular disease have been recently confirmed in rhesus macaques, in which disruption of endothelial cells *via* cytokine was observed (178).

A recently proposed microclot model suggests that small clots in the blood capillaries prevent oxygen from reaching the tissues and may cause Long COVID symptoms by limiting oxygen exchange. Plasma analysis of 70 patients with Long COVID confirmed the presence of microclots with platelet hyperactivation and resistance to fibrinolysis. Associated elevated α(2) antiplasmin and the acute inflammatory molecule Serum Amyloid A (SAA4) were observed in the supernatant and microclots (179). Twenty-four patients treated with an antiplatelet and antithrombotic regimen showed symptom improvement and reduced microclots (180).

### 3.13. Orthostatic tachycardia—Autonomic nervous system dysfunction

Autonomic dysfunction (dysautonomia) occurs in many Long COVID patients (181, 182). In one study, autonomic dysfunction occurred more commonly in those with neurologic symptoms (183). Dysautonomia is an autonomic nervous system (ANS) dysfunction, resulting in failure or overactivity of the ANS with alterations in blood pressure, breathing, GI tract motility, heart rate, and temperature regulation. Some Long COVID patients report orthostatic hypotension and/or postural orthostatic tachycardia (POTS) (184). POTS is a form of dysautonomia characterized by tachycardia within 10 min of standing up (>30 bpm increase from a supine position or > 120 bpm in the erect position) (185).

There is a long list of possible causes of dysautonomia. An interplay between the ANS and the immune system involves cytokines, which modulate the activity level of the sympathetic and parasympathetic nervous systems innervating multiple organs (186). POTS results from sympathetic nervous system dysfunction, and in one study was associated with increased IL-6 levels but not C-reactive protein (187).

Involvement of the brainstem and inferior frontal - olfactory regions in COVID-19 has been documented at autopsy and imaging studies (188). Many of the diverse symptoms experienced by Long COVID patients—including POTS—can be explained through the involvement of brainstem nuclei and tracts (189) which may reflect direct viral invasion, or indirect effects of neuroinflammation triggered by peripheral or central cytokine release, or effect of microclots.

An intriguing association between dysautonomia and antiphospholipid syndrome (APS, aka Hughes syndrome or “sticky blood”) has been observed (190). In addition to dysautonomia, APS is associated with a host of cardiac and neurologic manifestations. Both POTS and APS involve inflammatory cytokines (191) and can be triggered by viral infections, stress, and pregnancy. In Long COVID patients, autonomic dysfunction and some neurologic symptoms could be due to “sticky blood” and microclots resistant to fibrinolysis.

Proteomic analysis of POTS confirmed increased proteins involved in thrombogenicity and platelet activity, inflammation, cardiac contractility, skeletal muscle hypertrophy, and increased adrenergic activity (192). This proteomic footprint defines POTS, in part, as a hypercoagulable and proinflammatory state, mechanisms central to COVID-19 pathogenesis.

The occurrence of POTS after COVID-19 vaccination has been confirmed, although the incidence is five times less than that following COVID-19 infection (182, 193–195).

## 4. Loss of regulation of cytokine pathways in Long COVID patients

The protracted Long COVID clinical course could reflect impaired viral clearance with a reservoir of undetected viruses (196, 197). Residual viral fragments could be inciting inflammation. A recent autopsy study showed SARS-COV-2 RNA in multiple extrapulmonary anatomic sites for up to 230 days (198). Alternatively, viral reinfection could be occurring. Finally, Long COVID symptoms may reflect underlying end organ disease to the lungs, heart and other organs.

For Long COVID patients, it is more likely that the virus has been cleared and that the ongoing relapsing symptoms represent a continuing exaggerated response to cytokine release (199). Indeed, the majority of reported Long COVID symptoms can be directly attributed to cytokines. We propose that these patients exhibit an exaggerated response to the ongoing release of cytokines, which is key to understanding their disease’s pathophysiology.

Multiple checkpoint regulation sites with feedback inhibition are involved in the normal cytokine signaling pathways, allowing the cells to return to a quiescent, non-inflamed state. Autoinflammatory and autoimmune diseases can be caused by dysregulation and chronic activation of cytokine signaling (200). Dysregulation of cytokine inflammation may be occurring on a system or cellular level or a combination of both.

In patients with severe acute COVID-19, a systemic inflammatory response initiated by Acute Lung Injury (SIRS-ALI) can produce an inflammatory loop, resulting in cytokine release from numerous sources. Infection results in macrophage activation and release of inflammatory cytokines. These cytokines, IL-6, TNF, and IL-1β, promote the release of cell adhesion molecules (CAMS) and vascular endothelial growth factor in the lungs, reducing the barrier protection provided by the endothelium. Their release into the blood stimulates production and release of immature granulocytes, which can increase lung inflammation, leading to ARDS. These cytokines augment direct viral damage and persist long after the virus is cleared (201). However, since Long COVID patients do not typically present with severe pulmonary infections, this model may not directly pertain to the initiation or persistence of their inflammatory response.

### 4.1. System-level dysregulation of cytokine expression

Bidirectional communication between the peripheral immune system and the hypothalamic–pituitary–adrenal axis (HPA) modulates the inflammatory response. Cytokines cross the blood–brain barrier and activate the HPA axis, resulting in increased glucocorticoid secretion by the adrenal gland. Glucocorticoids, in turn, negatively feedback onto immune cells, inhibiting the expression of proinflammatory cytokines and increasing anti-inflammatory cytokines, thereby protecting the host from harmful effects of an overactive immune response. Impairments in HPA axis activity and associated hypocortisolism can result in chronic inflammatory or autoimmune diseases (202–205).

Viral-induced suppression and disruption of the host adrenal cortical response could result in transient hypoadrenalism, blocking the needed adrenal stress response (206, 207).

Notably, 39% of SARS survivors demonstrated transient hypocortisolism, developing several weeks after symptom onset and usually resolving by 1 year (208). Proposed mechanisms include hypophysitis, direct hypothalamic involvement with binding to ACE2 receptors, and mimicry with host antibodies directed against ACTH (203). Autopsy also showed adrenal gland necrosis (209) which could be secondary to direct adrenal gland infection or an autoimmune process. While intriguing, there is no correlation between SARS survivors with hypocortisolism and those who experience chronic fatigue.

In a recent study of 215 individuals, immune profiling confirmed cortisol levels approximately 50% lower among participants with Long COVID relative to matched control groups. Persistently decreased cortisol production in participants with Long COVID was observed for more than a year following acute infection (31). Lack of a rise in ACTH levels in these patients indicated a blunted compensatory response of the HPA. The same study also demonstrated exhausted T-cells, suggesting an ongoing inflammatory response.

### 4.2. Cellular level dysregulation of cytokine gene expression (P38 MAPK controlling pathway, polymorphisms, transcription factors, and epigenetics)

The severity and chronicity of COVID-19 symptoms may be attributed to different patterns of immunopathology (210, 211). Chronic disease was characterized by normal effector T cells pro-inflammatory response but without the normal recruitment signals to attract activated T cells (211).

On a cellular level, viruses can induce cytokine dysregulation through several mechanisms, including viral-induced epigenetic changes in gene expression, transcription factors, superimposed on underlying gene polymorphisms.

If cytokines are responsible for the myriad of chronic relapsing symptoms of the Long COVID patients, it is essential to understand the cellular controlling mechanisms that initiate or suppress cytokine release. Multiple signal transduction pathways control cytokine gene expression. The Janus kinase (JAK)-signal transducer and activator of transcription (STAT) pathway (212), and the p38 mitogen-activated protein kinases (MAK) play a central role in coordinating the immune system. Through phosphorylation, these pathways respond to cytokines and other external stimuli to control cytokine gene expression.

#### 4.2.1. p38 MAP kinase (p38 MAPK) regulation of proinflammatory cytokines

**Figure 4:**
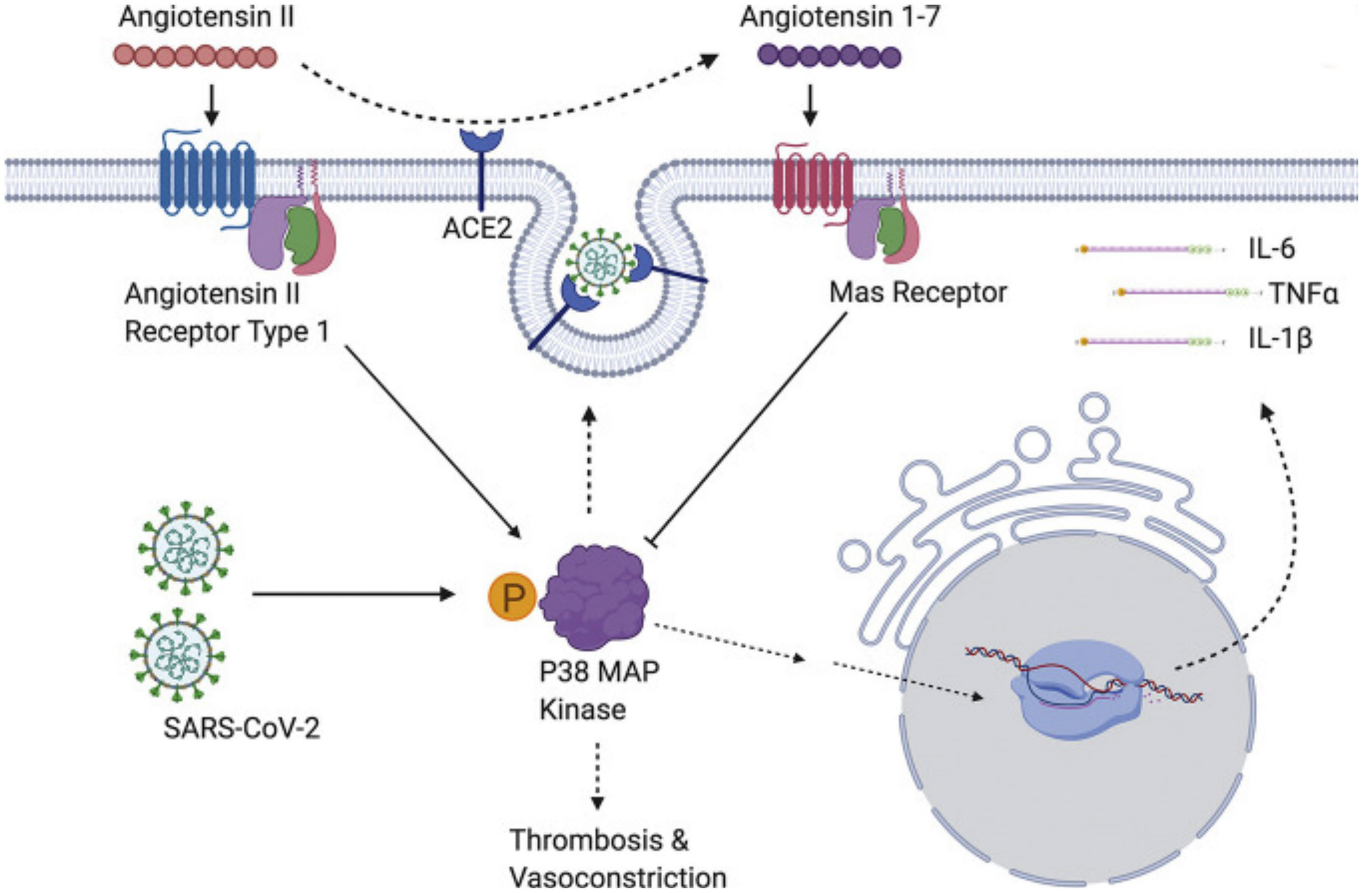
SARS-CoV-2 induces overwhelming inflammation by directly activating p38 and downregulating the Ang1-7 inhibitory pathway. Reproduced from “The role of antioxidants in the chemistry of oxidative stress: a review” by AM Pisoschi and A Pop. (http://creativecommons.org/licenses/by/4.0/).

The p38 mitogen-activated protein kinases serve as essential components of an intracellular signaling pathway, essential for immune function and inflammation. P38 responds to external stresses such as infection, thermal shock, osmotic shock, and ultraviolet radiation, activating downstream targets, including several kinases, transcription factors, and cytosolic proteins. The activated transcription factor NF-kB upregulates cytokine genes, producing an inflammatory response (213). In a positive feedback loop, cytokines then activate the same p38 MAPK protein. The P38 MAPK pathway provides critical regulation over the transcription and translation of pro-inflammatory cytokine genes (214, 215) (Figure 4).

Regulating the p38 MAPK pathway prevents runaway inflammation. Downregulation of p38 can occur through direct dephosphorylation (217). Downstream mitogen-activated kinases MK2 and MK3 play essential roles in regulating p38 MAPK (218). Genetically modified mice with “knockouts” of p38 MAPK or its downstream substrates MK2 and MK3 provide insights into these protein kinases’ role in disease models. In a mouse model with pemphigus vulgaris, inhibitors of p38 MAPK turned off cytokine release and prevented the blistering characteristic of the skin disease (219).

In an autoimmune encephalomyelitis model, MK2 exerts marked anti-inflammatory effects on p38 MAPK signaling. In MK2 knockout mice, in which the normal inhibitory function of the MK2 on p38 MAPK is blocked, there was a severe worsening of the clinical course (220).

The SARS-CoV virus causes p38 MAPK phosphorylation in infected cells, thereby activating physiological intracellular signaling cascades (221). Spike-mediated entry of SARS-CoV-2 triggers activation of p38 MAPK, occurring within the first 15–75 min after viral infection (222). SARS-CoV-2 directly upregulates p38 MAPK genes, rewiring host cell phosphorylations in 97 of 518 human kinases. This results in p38 MAPK activation with subsequent production of various cytokines, and the shutdown of mitotic kinases, producing cell cycle arrest (223). By potentiating the p38 pathway with gene upregulation and blocking p38 MAPK inhibitors, SARS-CoV-2 increases cytokine production, resulting in worsening inflammation and disease progression (216, 221). Conversely, inhibition of p38 signaling curtails the SARS-CoV-2 inflammatory response (222).

#### 4.2.2. Modification of gene expression through transcription factors and epigenetics

Cytokine gene expression is controlled by a network of transcription factors, including Nuclear Factor-kB (NF-κB), which is upregulated by SARS-CoV-2 (224, 225). NF-κB induces the expression of hundreds of proinflammatory and immune genes encoding for cytokines and chemokines and is a central controlling pathway for inflammation (224). The p38 MAPK pathway regulates the transcriptional activity of NF-κB through phosphorylation (213).

**Figure 5:**
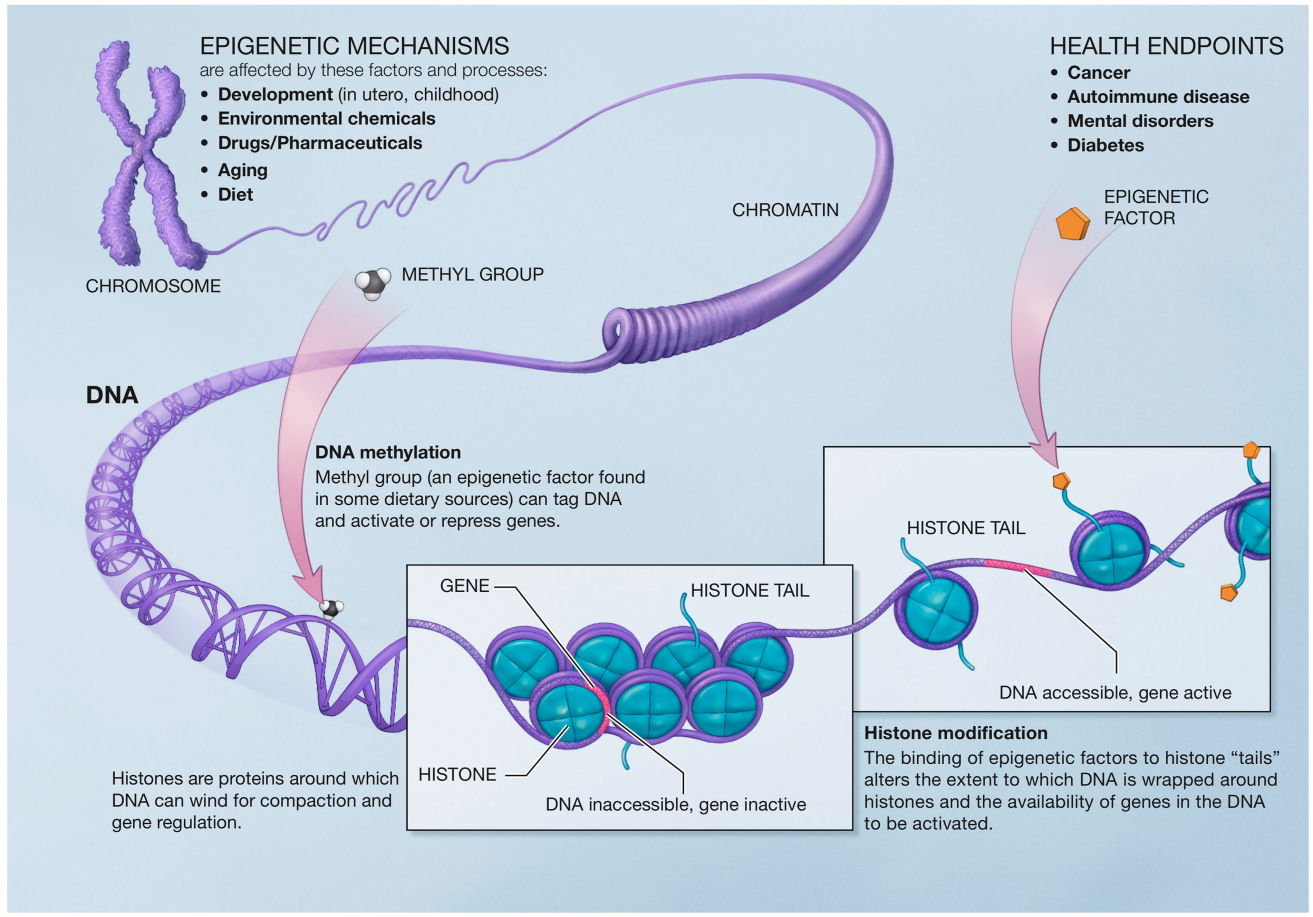
Epigenetic mechanisms altering gene expression. Reproduced from “A Scientific Illustration of How Epigenetic Mechanisms can Affect Health.” National Institutes of Health. (Image available for public use).

Epigenetics describes the genetic and non-genetic factors that control or modify gene expression without altering the underlying DNA code. These external or environmental factors interact with the host DNA, leading to phenotypic variation. Epigenetics mechanisms include DNA methylation, histone modifications, and chromatin remodeling (226) (Figure 5).

Epigenetics and transcriptional factors shape the adaptive immune response and provide a model to understand the genetic basis for the broad clinical spectrum encountered with SARS-CoV-2 infections (227, 228).

Coronavirus pathophysiology and disease severity are closely linked to epigenetic mechanisms, promoting viral entry, replication, evasion of host defenses, and immune hyperactivation (228, 229). There are remarkably 464 upregulated and 222 downregulated genes for the SARS-CoV-2 infection in the complex interaction between host and virus (230).

Transcriptional factors and viral epigenetic modification of host DNA expression result in loss of normal cytokine release regulation (226, 231). SARS-CoV-2 upregulates the p38 MAPK gene through phosphorylation, resulting in the production of a diverse spectrum of cytokines (223). These modifications can lead to increased inflammatory gene expression or loss of inhibition of the p38 MAPK signaling pathway.

Epigenomic analysis of blood hematopoietic and stem progenitor cells (HSPC) revealed sustained changes in hematopoiesis and innate immunity after COVID-19. These authors suggested that “this epigenetic reprogramming of HSPC may underlie long-term altered immune function following infection that are conveyed, through differentiation, to progeny innate immune cells.” (232).

#### 4.2.3. Genetic polymorphisms determine differences in symptoms and clinical severity

Genetic polymorphism in the cytokine genes’ regulatory regions can explain interindividual differences in the severity and symptoms of COVID-19. Different alleles of the cytokine genes affect the level and ratio of cytokines produced, altering the autoimmune and inflammatory response (233). ACE2 polymorphism is associated with susceptibility for SARS—CoV-2 infection and possibly the severity of disease (234, 235).

COVID-19 comorbidities, hypertension, diabetes mellitus, and coronary artery disease result in a more severe clinical course with acute SARS CoV-2 infection. Polymorphisms in 11 genes associated with these comorbidities, involved in regulating immune response, cytokine activity, and viral infection, can explain variations in susceptibility and disease severity (236).

Long COVID post-viral syndrome may be related to genetic predisposition. Single nucleotide polymorphisms of cytokine genes IL-6, TNF-α, IFN-γ, and IL-10 correlate with post-viral fatigue, pain, neurocognitive difficulties, and mood disturbances, respectively. Increased fatigue post-infection is associated with the T allele of IFN-γ +874 T/A SNP (237). Overall severity and duration of post-viral symptoms are associated with polymorphisms in the IFN-γ and IL-10 genes (238).

Polymorphisms may relate to the neurologic post-viral symptoms in Long COVID. For example, a recent study (239) focused on rs2106809, a SNP of the ACE2 promoter. The authors found that allele G of rs2106809 correlated with significantly higher ACE2 expression in the brain, and hypothesized that this SNP could be involved in Long COVID symptoms such as brain fog. However, further studies specifically involving Long COVID patients would be needed to test this hypothesis, and to identify other potential genetic contributions to Long COVID. Another study (240) found that Post-COVID pain was not associated with inflammatory polymorphisms.

### 4.3. Other factors contributing to the cytokine release syndrome

#### 4.3.1. Oxidative stress

An imbalance between production and accumulation of reactive oxygen species (ROS) causes oxidative stress. When oxygen-free radicals exceed antioxidant capacity, OS can trigger inflammation or result from cytokine release and inflammation (241). OS and CRS are closely related and interdependent processes. Positive feedback loops between OS and CRS result in amplifying the clinical effect and a potentially vicious cycle of OS and inflammation (242, 243). For chronic diseases, low-grade inflammation and OS are tightly linked and interdependent processes (243). The signaling link between OS and cytokines involves the p38 MAPK (244, 245). Combined antioxidant and anti-inflammatory therapy provides an intriguing two-pronged approach to treating the chronic inflammation of the Long COVID patients (246, 247).

#### 4.3.2. Bioenergetic abnormalities

Alterations in cellular energy metabolism from aberrant mitochondrial function may result in insufficient ATP generation (248, 249). Proinflammatory cytokines and OS potentiate the mitochondrial dysfunction in ME/CFS, resulting in glucose hypometabolism and decreased ATP (125). Blockage of the TCA cycle, leading to less efficient metabolism, has been proposed (250). Mitochondria provide the metabolites essential for chromatin remodeling and DNA methylation in epigenetic DNA modifications (251). Dysfunctional mitochondria also contribute to microglial activation and neuroinflammation (252) and to central and peripheral fatigue.

Early studies suggested that SARS-CoV-2 hijacks the host cell mitochondria where it replicates, impairs mitochondrial dynamics and leads to cell death, while promoting its own survival (123, 253). Translocation of viral RNA, RNA transcripts, and proteins into the host mitochondria results in inflammasome activation and suppression of host innate and adaptive immunity (123, 253). Experimental studies in COVID-19 patients have confirmed mitochondrial dysfunction and metabolic changes with increased inefficient glycolysis (254, 255). With increasing age, a decline in ACE2 function and mitochondrial function may make older patients more susceptible to viral induced mitochondrial dysfunction and SARS CoV-2 infection (123).

Long COVID exercise limitation may be due to dysfunctional metabolism and bioenergetic abnormalities. Reduced peak oxygen consumption is observed in studies of post-COVID patients independent of cardiopulmonary status (123, 256, 257) with associated reduced oxygen extraction (257).

## 5. Experience from post SARS patients who experience chronic fatigue syndrome

Following other epidemics, including the 2003 SARS epidemic, a group of patients showed symptoms of chronic fatigue and sleep disturbances, very similar to those of the Long COVID patients (112, 258). Follow up of 233 survivors at 4 years showed 40.3% with ongoing chronic fatigue, with 27.1% meeting the modified 1994 CDC criteria for CFS. In personal communication with the author regarding ongoing follow up of these patients, Dr. Lam indicated that 100 patients were being followed for chronic fatigue at 4 years. Now, 18 years after the SARS outbreak, 70 patients showed interval resolution of symptoms, while 30 (13%) progressed to chronic illness with ongoing fatigue and sleep disturbance. A few patients with hypocortisolism responded to steroid replacement therapy. In the majority, there was no correlation between fatigue severity and salivary cortisol levels.

## 6. Progression to ME/CFS and the role of autoimmunity

Why some patients with post-viral syndrome progress to ME/CFS is a critical issue that can involve polymorphic genetic predisposition, an environmental or infectious trigger, and immune system dysregulation (259).

Autoantibodies play an important role in the COVID-19 pathophysiology (260) with dramatically increased autoantibodies compared to non-infected individuals. The wide spectrum of clinical presentations of COVID-19 may relate to altered and misdirected immune response. Whether this explains Long COVID symptoms and persistence has not been established.

However, chronic systemic inflammation leads to a host of illnesses, including autoimmune diseases (261). Since the autoimmune model for post-infectious ME/CFS is increasingly recognized (261–263), this could be a determining factor in the progression from post-viral syndrome to ME/CFS.

The viral triggering of autoimmunity is intimately associated with cytokines (195) and epigenetic alterations of gene expression (264). It is known that coronaviruses trigger autoimmunity. The SARS-CoV virus-induced autoantibodies against lung epithelial cells (265). SARS-CoV-2 infection has precipitated a host of autoimmune diseases, including multisystem inflammatory syndrome in children (MIS-C) (259, 266).

For the Long COVID patients, continuing cytokine expression can produce a low-grade chronic inflammatory state, which can further lead to activated, dysregulated microglia and neuroinflammation (267). A working hypothesis is that some patients will have a genetic polymorphism predisposing them to autoimmunity. Epigenetic changes in gene expression induced by the virus or other external factors may provide the trigger that initiates the progression to autoimmunity and ME/CFS. Recent reports confirm the development of ME/CFS in a subset of Long COVID patients with mild to moderate initial acute symptoms. Biomarkers suggest ongoing inflammation and hypoperfusion as pathomechanisms (268). In patients with unexplained dyspnea, cardiopulmonary testing confirmed circulatory impairment, abnormal ventilatory pattern and ME/CFS in Long COVID patients (269).

## 7. Therapeutic implications

### 7.1. Severe acute COVID

The use of anti-cytokine / anti-inflammatory therapies in patients with severe acute COVID-19 has been investigated (270). IL-1 blockers in COVID-19 include anakinra, canakinumab, and rilonacept. In a phase3 trial, administration of Anakinra increased full recovery and lowered mortality compared to standard care alone. On day 28, 50.4% of the anakinra group was fully recovered compared with 26.5% of the standard care group (*p* < 0.0.0001). Mortality was 6.9% with standard care and 3.2% with anakinra (271).

IL-6 blockers include tocilizumab, sarilumab, and siltuximab. One single institution study of tocilizumab in serve COVID-19 found no difference in mortality compared to the control group (272). In another study, tocilizumab administration decreased the likelihood of progression to mechanical ventilation or death, but did not enhance survival in non-ventilated patients (273).

Other anti-cytokine therapies in severe COVID-19 include tumor necrosis factor inhibitors. In a meta-analysis of 84 studies describing use of anti-TNF treatments it was concluded that TNF-α inhibitors are associated with a lower probability of hospitalization and severe COVID-19 when compared to any other treatment for an underlying inflammatory disease (274).

Glucocorticoids, producing a broad-spectrum immune suppression, have been widely used in severe acute COVID-19. The RECOVERY trial found a reduction in mortality amongst mechanically ventilated patients (29.3% vs. 41.4%) for those patients receiving dexamethasone (275).

### 7.2. Long COVID

Large national clinical trials include the RECOVER^1^ in the United States and STIMULATE-ICP^2^ in the United Kingdom. The 1.5 billion dollar RECOVER trial will test more than a dozen immunosuppressants, immune enhancing drugs, corticosteroids, and antiviral drugs to correct the immune-inflammatory dysfunction of Long COVID. The STIMULATE-ICP trial will test several medicines against Long COVID including the anti-inflammatory colchicine, two antihistamines famotidine and loratadine, and an anti-clotting drug, rivaroxaban.

For Long COVID, results of therapeutic studies are so far limited. In a recent review of pharmacologic treatment of Long COVID, Chee et al. reported on six published trials and 54 ongoing clinical trials (276). Study heterogeneity and varying definitions of “Long COVID” limit comparisons and attempts to draw conclusions.

Chronic inflammation and endothelial dysfunction was investigated in a study of Sulodexide, a novel agent that attenuates endothelial thrombosis and inflammation (277). Following a three-week course of Sulodexide, there was a significant improvement in endothelial function with alleviation of chest pain and palpitations (277). Addressing the close interplay between inflammation, endothelial dysfunction and thrombosis, ongoing studies are evaluating the use of low-dose statins, apixaban and atorvastatin, omega-3 fish oil (276, 278).

Chronic inflammation and pulmonary fibrosis was studied with therapeutic agents pirfenidone (279, 280), corticosteroids (281), and montelukast (282) for treatment of respiratory symptoms of Long COVID. In an open-label study evaluating the utility of prednisolone in patients with post COVID-19 parenchymal lung abnormalities, the administration of prednisolone 10 mg daily for 6 weeks was associated with an improvement in dyspnea and respiratory function (283). In another study, a short, four-day course of corticosteroids reduced symptoms and immunological alterations underlying Long COVID (284).

The neurocognitive symptoms of Long COVID are being studied with various pharmacologic agents including atorvastatin, vortioxetine, fampridine, RUCONEST, and intravenous gamma globulin, as well as nasal sprays containing ivermectin, and retinoic acid.

Increased oxidative stress in Long COVID patients with immune dysregulation is being evaluated with repurposing nutraceuticals and Vitamin supplements (Vitamin C and Vitamin D) as potential therapeutic strategies. Gut dysbiosis and its role in the chronic inflammation of Long COVID patients is being studied with probiotics as a treatment regimen for the treatment and management of Long COVID.

There is currently insufficient data on which to base meaningful conclusions regarding therapeutic interventions for Long COVID. There does seem to be anecdotal experience that “resetting” the immune-inflammatory system can have at least short-term benefits and relief of some symptoms. In this vein, it was observed that some patients experienced symptom relief following COVID-19 vaccination. In 11 studies evaluating the impact of vaccines on patients with Long COVID, seven found improvement in ongoing symptoms while four found no change or even worsening of symptoms (163).

There is anecdotal evidence of similar improvement in Long COVID symptoms with pregnancy, which results in major alterations in the immune-inflammatory system. This is in line with previous reports on remission of Multiple Sclerosis (285), Rheumatoid Arthritis (285, 286), and Autoimmune Hepatitis (287) during pregnancy, suggesting immunosuppressive effects of pregnancy (288). The multifaceted and complex nature of Long COVID with different subgroups and distinct pathophysiologies may require a combination of therapies, tailored to the underlying cause and individual patient symptoms. Returning the body to its homeostasis with a combination of anti-inflammatory, antihistamines, and drugs that treat blood vessel inflammation and microclots may yield results (289).

Ultimately, the prevention of Long COVID is an equally important topic to be addressed. The effect of vaccinations in decreasing Long COVID has been discussed above. Modulating and decreasing the initial immune inflammatory response in COVID-19 may be beneficial in reducing the incidence of Long COVID. A recent preprint indicated a 42% relative decrease (6.3% vs. 10.6%) in the incidence of Long COVID in participants who received early outpatient COVID-19 treatment with the drug metformin with known anti-inflammatory effects (290) compared to a matched placebo control group (291).

## 8. Final thoughts

The proposed explanation of the pathophysiology of the persistent Long COVID symptoms is likely incomplete. However, it provides a reasonable model to consider as part of the pathophysiology of the plight of the millions of persons who are stuck in a seemingly never-ending recovery from COVID-19. It is also important to note that cytokines do not have to be detectable in the periphery to have an effect on immune dysregulation and its possible downstream consequences (292). Moreover, methodological limitations can impact accuracy of cytokine measurements (29). Therefore, it remains a challenge to use peripheral blood cytokines measures as easily accessible biomarkers of the disease (29).

The seemingly competing mechanisms of inflammation and microthrombosis may actually be two sides of the same immunothrombotic coin. Both processes can occur and are intimately interrelated with one leading to the other. However, it is conceivable that in some Long COVID patients, one or the other processes may predominate, leading to different symptoms. This also has important implications for potential treatment options for different Long COVID patients directed at inflammation vs. clotting pathways or both.

We hope this article sheds light on how the science of cytokines and their controlling signaling pathways can explain a multitude of diverse Long COVID symptoms. Common underlying processes are likely involved in the pathophysiology of Long COVID and other chronic diseases and post-viral syndromes. Understanding this pathophysiology should help us to define and test therapeutic interventions, opening the pathway to recovery.

## Summary

Dysregulated immune-inflammatory response with elevated peripheral and central cytokines can explain Long COVID symptoms. Hyperreactive brain microglia can modulate a host of CNS-mediated symptoms.

## Data availability statement

The original contributions presented in the study are included in the article/supplementary material, further inquiries can be directed to the corresponding author.

## Author contributions

RNL, RJL, and AA contributed to the conception and design of the study. RNL wrote the first draft of the manuscript. RNL, RJL, and AA wrote sections of the manuscript. All authors contributed to the article and approved the submitted version.

## Conflict of interest

The authors declare that the research was conducted in the absence of any commercial or financial relationships that could be construed as a potential conflict of interest.

## Publisher’s note

All claims expressed in this article are solely those of the authors and do not necessarily represent those of their affiliated organizations, or those of the publisher, the editors and the reviewers. Any product that may be evaluated in this article, or claim that may be made by its manufacturer, is not guaranteed or endorsed by the publisher.

## Footnotes

^1^ https://recovercovid.org

^2^ https://www.stimulate-icp.org
